# Supplementary material for: A potent neutralizing nanobody against SARS‐CoV‐2 with inhaled delivery potential
Source: MedComm (2020). 2021 Mar 4;2(1):101–13. doi: 10.1002/mco2.60 (PMC8013425; doi:10.1002/mco2.60)
Supplement: Supplementary file 1 — Supporting Information [file MCO2-2-101-s001.pdf]

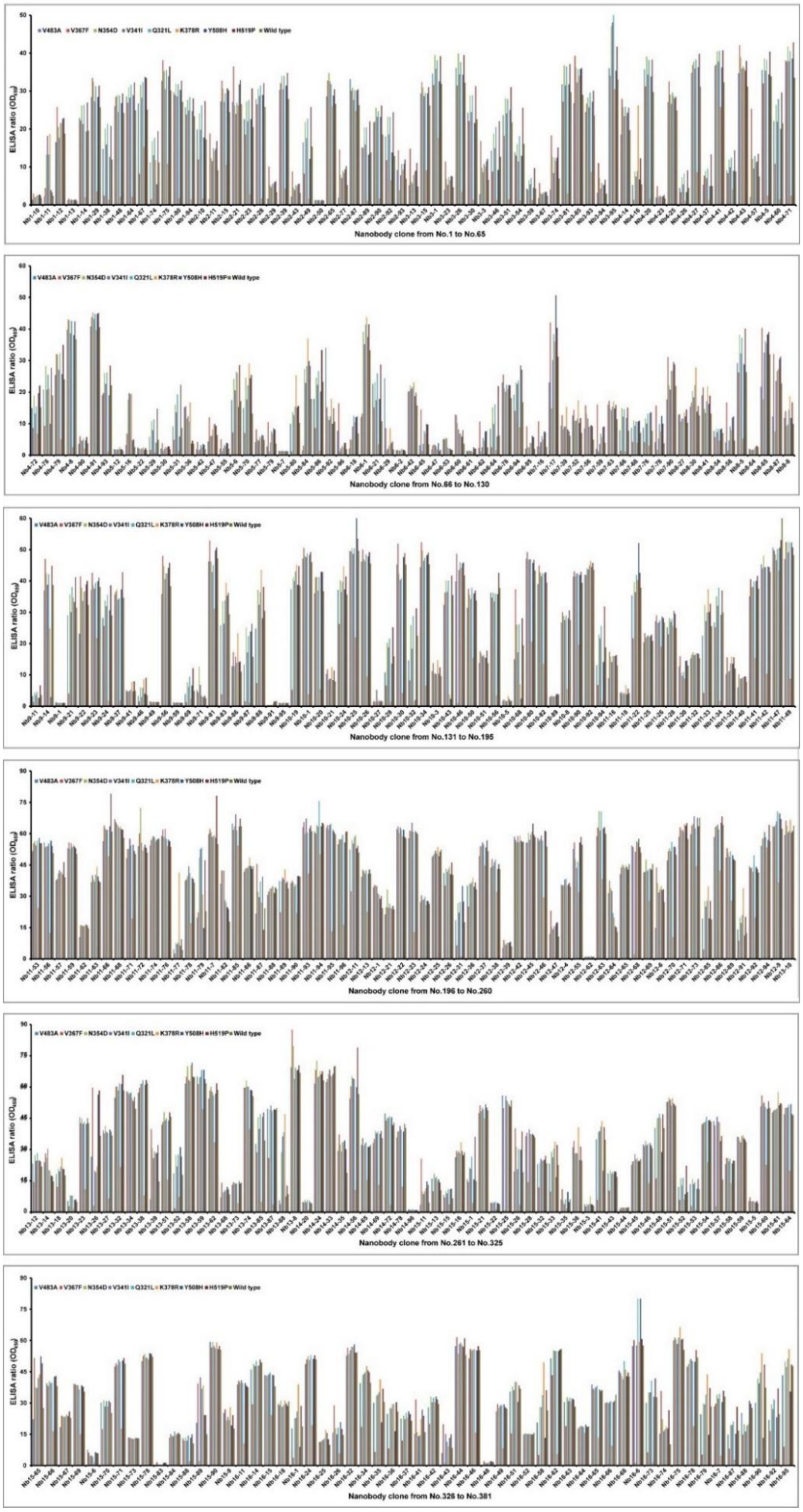

Supplementary Figure 1. The binding activity of positive colonies to different SARS-CoV-2-RBD mutants.

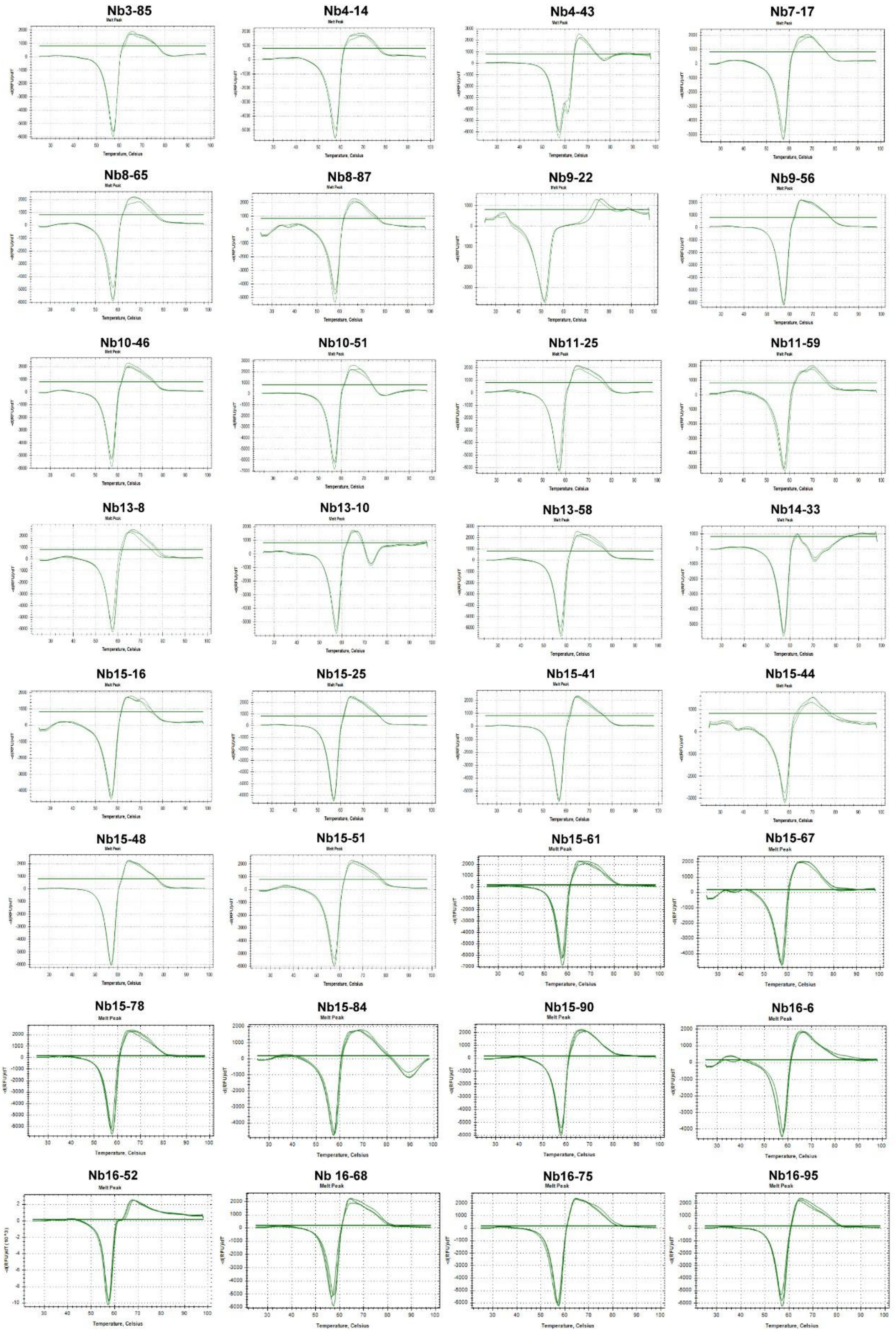

Supplementary Figure 2. The Tm value detection of the 32 purified SARS-CoV-2-RBD specific Nbs.
